# Allosteric Inhibitors of NMDA Receptor Functions

**DOI:** 10.3390/ph3103240

**Published:** 2010-10-14

**Authors:** Gabriela K. Popescu, Swetha Murthy, William F. Borschel

**Affiliations:** Department of Biochemistry, University at Buffalo, SUNY/3435 Main Street, Buffalo NY 14214, USA; E-Mails: smurthy2@buffalo.edu (S.M.); wfb3@buffalo.edu (W.F.B.)

**Keywords:** NMDA receptor, allosteric modulation, gating modifiers

## Abstract

NMDA receptors are glutamate-activated ion-channels involved in many essential brain functions including learning, memory, cognition, and behavior. Given this broad range of function it is not surprising that the initial attempts to correct NMDA receptor-mediated pathologies with en-mass receptor blockade were derailed by unacceptable side effects. Recent successes with milder or more targeted pharmaceuticals and increasing knowledge of how these receptors operate offer new incentives for rational development of effective NMDA receptor-targeted therapies. In this article we review evidence that l-alanine, a glycine-site partial agonist and pregnanolone sulfate, a use-dependent allosteric inhibitor, while attenuating NMDA receptor activity to similar levels elicit remarkably dissimilar functional outcomes. We suggest that detailed understanding of NMDA receptor activation mechanisms and of structural correlates of function will help better match modulator with function and neurological condition and may unleash the yet untapped potential of NMDA receptor pharmaceutics.

## 1. Introduction

In the still mysterious puzzle of brain function, the NMDA receptors play vital roles in the transmission, integration and plasticity of excitatory signals. In these capacities, the NMDA receptors are essential for higher functions of the mammalian central nervous system but can also be perpetrators or accomplices in the initiation and development of many pernicious neuropathies [1,2,3]. Pharmaceuticals that control NMDA receptor functions may be enormously beneficial in the prevention, therapy or management of many afflictions including acute and chronic neurodegeneration, mental retardation, schizophrenia, epilepsy, addiction and pain [4,5,6,7,8]. However, this much anticipated therapeutic potential is still largely unrealized. Furthermore, the severe side effects of first-generation NMDA receptor blockers and disappointing results from many clinical trials that failed to show neuroprotective efficacy may cause future drug development efforts to be side-tracked or even abandoned. In this article, we argue that the hidden treasure of NMDA receptor-targeted therapies is within reach and the key to their success lies in the better understanding of how the receptor works, followed with rational development of medicinal allosteric modulators. 

## 2. NMDA Receptor Modulators as Potential Therapies

NMDA receptors are glutamate-gated ion channels with high calcium permeability. They are widely expressed throughout brain and spinal cord and are enriched in the post-synaptic membranes of excitatory synapses [9,10]. Many aspects of these receptors’ contribution to brain physiology and pathology are insufficiently understood and represent the subject of vigorous investigation. Despite many uncertainties, it is widely accepted that critical aspects of their physiological function are: to detect coincident activation of the pre- and post-synaptic neurons; to integrate this synchronous activation over time and with overlapping environmental cues; and to translate this complex information into post-synaptic calcium transients. Both the amount and the time course of the calcium influx represent compelling biochemical signals which set off specific chains of physiologic or pathologic processes. 

During normal development, NMDA receptor-mediated calcium fluxes are necessary for the formation and plasticity of synaptic connections, but also to initiate apoptotic pathways that mediate physiologic synaptic pruning. Disorders at this level have been implicated with schizophrenia, cognitive disabilities and mood disorders [4,7,11,12,13]. Ongoing NMDA receptor signaling remains critical in adult, and malfunctions underlie many brain disorders: over activation of NMDA receptors can cause epileptic seizures or excitotoxic neurodegeneration; insufficient activation underlies some forms of psychoses and cognitive deficits; and anomalous plasticity has been implicated in several forms of addiction, neuropathic pain, and behavioral disorders [8,14,15,16]. Thus modulators of NMDA receptor activity, whether they attenuate or boost receptor-mediated fluxes, may represent valuable pharmaceuticals for both developmental and adult onset pathologies [17,18]. 

Many physiologic and pharmacologic ligands bind directly to NMDA receptor side-chains and change the amplitude and time course of the glutamate-triggered calcium-fluxes. By their mechanism of action, these ligands can be divided into two broad categories: pore blockers and allosteric modulators. 

Blockers bind to residues located close to the channel pore and reduce receptor-mediated currents by physically obstructing the permeation pathway [19,20,21]. Within this category, open-channel blockers can access their binding sites only after the channel opens. The level of the resulting inhibition depends on blocker concentration, competing ions, channel affinity for the blocker, and channel kinetics. In addition, because blocker binding-sites are often located within the membrane field, the blocker binding-rate is voltage-dependent [22]. 

Many aspects of NMDA receptor modulation by blockers are insufficiently understood, despite their clear physiological impact and pharmacological potential. Magnesium ions are physiologic blockers which impart voltage-sensitivity to NMDA receptor currents [19,20]. NMDA receptors detect coincident activation of presynaptic and postsynaptic cells, by passing current only when glutamate is present in the cleft, to initiate channel gating, and the post-synaptic membrane is depolarized, to allow relief from Mg^2+^ block. Ketamine, MK-801 and dexomethorphane are examples of voltage-dependent open-channel blockers that are investigated as potential therapies [1]. Memantine, a low-affinity blocker, is already in use for moderate to severe Alzheimer’s dementia and is examined for potential benefits in patients suffering from neuropathic pain, macular degeneration, mood disorders and other neurologic conditions [22,23,24]. 

In contrast to blockers, allosteric modulators change receptor-mediated currents by altering the receptor’s activation reaction. Commonly, the term allosteric modulator is used to describe small diffusible molecules that upon binding to receptor sites shift the rate with which the receptor’s built-in gating machinery operates. Allosteric modulation of NMDA receptors at extracellular sites has received prolific attention and several comprehensive reviews have been recently published [25,26,27,28]. Far less understood are allosteric modulators that bind within the transmembrane domain, including the pore, and within the intracellular domain. For example, evidence indicates that some blockers, aside from obstructing current flow through open channels also affect gating [22,29,30,31,32]. Gating is also controlled, either by direct binding or covalent modification by intracellular signaling molecules such as calmodulin, actinin and a variety of kinases and phosphatases [33,34,35,36,37,38]. Detailed characterization of NMDA receptor allosteric sites remains a desirable objective that awaits achievement.

Glutamate and glycine represent special cases of gating modifiers because in their absence channel openings occur with undetectably low probability. Thus functionally, glutamate and glycine are *de facto* required co-agonists. Physiologically however, glutamate and glycine carry specific biological information and their synaptic concentrations are controlled differently. Glutamate is the principal neurotransmitter in mammalian brain: it is released in a pulsatile fashion from a presynaptic bouton and informs the surroundings that the presynaptic neuron has fired. At some hippocampal synapses, the extracellular glutamate transient was estimated to reach 1 mM and to persist ~1 ms [39,40,41]. These values are consistent with saturation of glutamate-binding sites on NMDA receptors following the release of a single synaptic vesicle. In contrast, glycine has most likely a modulatory, albeit critical, role. Most probably it is continually present in the synaptic cleft and its ambient levels, controlled by neuronal and glial glycine transporters, can dictate the magnitude of NMDA receptor signals [42,43,44]. Competitive antagonists at the glutamate site, such as APV, disturb synaptic transmission profoundly and are incompatible with most therapeutic interventions. Conversely, glycine-site full agonists (D-serine), partial agonists (D-cycloserine, l-alanine) and antagonists have been used with promising results in a variety of disorders [45,46,47]. 

All other small molecules that interact with extracellular NMDA receptor residues and are noncompetitive with glycine or glutamate, modulate channel opening probability by communicating at a distance either with the agonist-binding sites to change their affinities, or with the gating machinery to change the receptor’s opening efficacy. For NMDA receptors, heterotropic modulators that can open the channel in the absence of agonists have not been described, thus the effects of allosteric modulators can be evaluated only relative to agonist-elicited currents. Recently, the development and characterization of NMDA receptor mutants that are constitutively open with respect to glycine or glutamate, may provide reagents that having fixed and maximal agonist affinities can be used to isolate experimentally gating effects of allosteric modulators [48,49]. 

From a drug development perspective, allosteric modulators present a number of important advantages compared to agonists, antagonists and blockers. First, because allosteric sites are saturable, the modulation has an upper limit, thus minimizing overdose risks. Second, allosteric modulators are only effective on endogenously-activated receptors, thus maintaining the natural rhythm of glutamatergic signaling. Last, because they bind to parts of receptors that are less stringently conserved than the channel pore or the agonist binding sites, they are more likely to exhibit isoform specificity [26]. To derive therapeutic gain from the rich pharmacology of NMDA receptors is necessary to have more detailed knowledge of the structural determinants of allosteric sites and the specific mechanism by which these control receptor functions.

## 3. Structural Information of NMDA Receptor Allosteric Sites Is Limited

**Figure 1:**
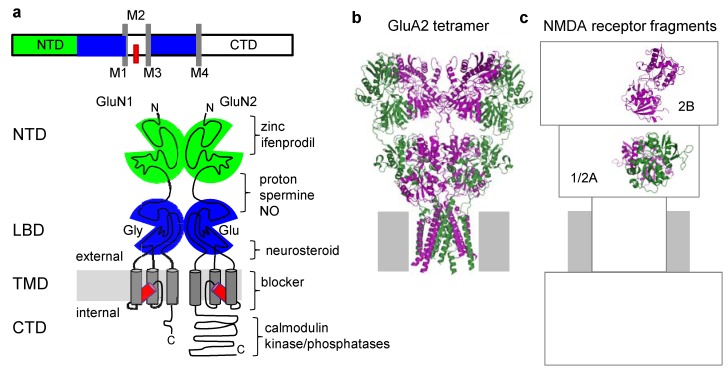
Molecular architecture of NMDA receptors. (a) Topology of a generic glutamate receptor subunit *(top)* and modular organization of GluN1 and GluN2 subunits (*bottom*). (b) Structural model of the homotetrameric GluA2 receptor, a member of the AMPA-sensitive ionotropic glutamate receptor family (3KGC) [51]. (c) Structural models of NMDA receptors are limited to soluble fragments of GluN2B-NTD (3JPY) [53] and of GluN1 and GluN2A LBDs, including a LBD dimer (2A5T) [54]; direct information about structural organization of TMD and CTD is absent.

Glutamate-activated NMDA receptors are tetramers of two homologous glycine-binding GluN1 subunits and two glutamate-binding GluN2 subunits. In the ionotropic glutamate receptor family, all subunits have modular organization and similar topology [28]. The extracellular portion consists of two tandem globular domains: a distal N-terminal domain (NTD), and a membrane-proximal ligand-binding domain (LBD) (Figure 1a). Three flexible linkers connect the LBD to the transmembrane domain (TMD), which consists of three membrane-spanning helices (M1, M2 and M4) and a pore-lining re-entrant loop (M2). The intracellular portion consists largely of the C-terminal domain (CTD). Functional NMDA receptors assemble as dimers of GluN1/GluN2 heterodimers, but whether like-subunits are situated vicinal or diagonal to one another is unknown [50,51,52]. Given that inter-subunit interfaces may represent binding sites with allosteric potential it will be important to delineate the exact order of NMDA receptor subunits around the central pore and the atomic organization of inter-subunit and inter-module interfaces. 

NMDA receptors are expressed with substantial molecular diversity and the exact subunit composition of native receptors is unknown. Tetramers may assemble from eight GluN1 splice variants (1a-c, 2a-c and 3a, b) and four genetically encoded GluN2 isoforms (A-D). Studies with recombinant preparations have demonstrated that isoforms differ in their kinetics, functional role and also in their pharmacology [3,55,56]. Thus to develop NMDA receptor-targeted drugs that are effective and safe it will be necessary to know with atomic resolution the structures of biologically active isoforms and the detailed arrangement of residues within allosteric sites. 

Over the past year, the first high resolution description of the GluA2 tetramer, a member of the AMPA-sensitive glutamate receptor family, has marked a milestone in the investigation of ionotropic glutamate receptors [51]. Although the protein consists of four identical protomers, two types of conformers were observed. Two of the subunits, rendered green in Figure 1b, adopt straight conformations, with the NTD stacked onto the LBD of the same subunit and in alignment with the TMD. In contrast, the other two subunits, rendered purple, appear twisted, with the NTD switching from one dimer pair to the other. The arrangement of residues within extracellular modules is in good agreement with previously published structures of LBD and NTD fragments belonging to a series of glutamate receptor subunits. The resolution in the TMD prevented unambiguous residue assignments in the pore region. Initial evidence indicates that in NMDA receptors, GluN1 subunits may adopt straight conformations and the GluN2 subunits may cross over [51]. However, this and many other aspects of the NMDA receptor three-dimensional architecture are unknown and the available high resolution information is limited to isolated receptor modules. 

Structural details have been elucidated for the isolated LBD of GluN1 subunits [57,58]; for a dimer formed by the soluble LBDs of GluN1 and GluN2A subunits [54]; and for the isolated NTD of GluN2B subunit [53] (Figure 1c). These models reveal similar globular structures, each consisting of two mobile lobes connected by a flexible hinge. In all structures, the cleft between lobes cradles binding sites for ligands with allosteric effects on gating. Several allosteric binding sites were described with high resolution revealing the residues responsible for ligand affinity and specificity. These are: the GluN1-LBD in complex with the agonists glycine and d-serine, the partial agonists d-cycloserine, ACPC and ACBC, and with the antagonists cycloleucine and DCKA [57,58]; the GluN1-LBD/GluN2A-LBD dimer in complex with the agonists glycine and glutamate respectively [54]; and the GluN2B-NTD bound with Zn^2+^ [53] (Table 1). These modulatory sites have tremendous pharmacologic potential and represent the molecular targets for many of the drugs currently in preclinical trials [15,25]. 

**Table 1:** Allosteric sites of NMDA receptors with known structure.

Protein	Ligand	Pharmacological action	MMDB/PDB ID	Reference
GluN1-LBD	Glycine	co-agonist (neuromodulator)	23627/1PB7	[54,57]
			36073/2A5T	
	D-serine	co-agonist (neuromodulator)	23628/1PB8	[57]
	ACBC	partial agonist (synthetic)	34203/1Y1Z	[58]
	ACPC	partial agonist (synthetic)	34204/1Y20	[58]
	Cycloleucine	antagonist; (synthetic)	34202/1Y1M	[58]
	DCKA	antagonist; (synthetic)	24334/1PBQ	[57]
	DCS	partial agonist; (synthetic)	23629/1PB9	[57]
GluN2A-LBD	Glutamate	agonist (neurotransmitter)	36073/2A5S	[54]
			36074/2A5T	
GluN2B-NTD	Zinc	non competitive inhibitor (neuromodulator)	78606/3JPY	[53]

Unfortunately no structural information is yet available for several other important modulatory sites. The residues implicated in potentiation by polyamine are located on the NTD of GluN1 subunits, a module for which structural information is lacking [59]. Functional studies of NMDA receptor modulation by nitric oxide have implicated residues located in the linker joining the NTD and LBD of GluN2 subunits [60]. Given the apparent flexibility of the polypeptide chain in this region, it will be challenging, yet important, to obtain structural information for this particular modulatory site. Even less characterized are the sites responsible for proton inhibition and neurosteroid modulation [61,62,63,64]. These represent physiologic modulatory sites and have clear potential for drug development. Until high resolution structural models for these sites become available, efforts to reveal the mechanism by which allosteric modulators affect receptor functions will have to rely more heavily on complementary lines of investigation, including kinetic studies.

## 4. NMDA Receptors Have Multi-Step Gating Reactions

Among fast-acting neurotransmitter receptors, NMDA receptors are remarkably slow by ion channel standards. The macroscopic response reaches peak within tens of milliseconds after stimulation, clearly several milliseconds after the neurotransmitter glutamate has been cleared from the cleft. However, even if delayed in onset, the response is characteristically long-lived, lasting from tens of milliseconds to seconds, depending on the receptor isoform expressed and environmental cues [2,3]. Such protracted and long-lasting activation is well suited for the receptor’s function as synaptic integrator. From a practical point of view, it has permitted unprecedented insight in their step-wise activation mechanism [65,66]. 

Real-time observations of currents passed by individual NMDA receptors composed of GluN1-1a and GluN2A subunits (1/2A receptors) have provided valuable information about the order in which the transformations that make up the receptor’s activation pathway occur. This receptor isoform, abundantly expressed in adult brain and spinal cord, has relatively high conductance (50−70 pS) and high open probability (0.6). Thus, it allows recordings that have high signal-to-noise ratios and large numbers of openings, making it well suited for statistical analyses [65,67,68,69]. In the presence of saturating levels of glutamate (1 mM) and glycine (0.1 mM) the channel opens frequently generating seconds-long intense bursts of openings (active periods) separated by seconds-long periods when no openings are observed despite the continued presence of both co-agonists (desensitized periods). The fact that the closures and openings observed over an extended period of time can be sorted into several groups by their characteristic mean life-times is a clear indication that the receptors, whether open or closed, can exist in several conformations which differ in stability. 

A number of recent single-channel studies support the hypothesis that when fully occupied with glutamate and glycine, all NMDA receptor isoforms oscillate between five closed and two open states [66,67,68,69,70,71]. In kinetic parlance, a state represents a spectrum of conformations with similar free-energies that can be resolved kinetically in a current trace obtained from a single receptor. Theoretically, based on the number of subunits and structural modules in each subunit, and considering that these can adopt distinct positions relative to one another, the range of energetically distinct receptor conformations is surely much larger than five and two for closed- and open-channel receptors, respectively. Thus kinetic models derived from these types of measurements represent most likely the visible envelope of intramolecular movements rather than unitary activation steps, which remain hidden. Still, these models represent a valuable advancement in the effort to parse out the steps by which channels become active and improve considerably the resolution with which modulator-sensitive steps can be identified. 

Remarkably, despite their low resolution, these models account well for the entire range of observed single-channel behaviors [69,71,72]; they have been used successfully to reproduce the waveform of ensemble responses under a variety of conditions [73,74]; and modeling has predicted correctly previously unsuspected receptor properties and functions [75,76,77]. Also, in several instances, these models were instrumental in the better understanding of the mechanisms employed by individual allosteric modulators [73,74,78,79,80,81]. 

Importantly, for receptors with multi-step gating it may be possible to find allosteric modulators that act by modifying distinct isomerization steps and thus enforce distinct functional outcomes [82]. Given that to be well-tolerated, NMDA receptor inhibitors will have to permit normal synaptic transmission while reducing responses from pathologically activated receptors, a major effort has been to search for isoform specific modulators [9,83]. The possibility that even for the same receptor isoform a modulator may target preferentially responses to specific stimulation patterns represents an additional and very attractive layer of specificity. Furthermore, recent evidence points to distinct roles of synaptic and extrasynaptic receptors in neurodegeneration [84]. Because these two populations of receptors are subject to dissimilar patterns of glutamate exposure, drugs that will preferentially inhibit receptors chronically stimulated to glutamate (extrasynaptic) but spare those only briefly stimulated (synaptic) may have advantages over current approaches.

In the remainder of this article we illustrate the idea of mechanism-based modulation of specific functions by reviewing previously published results from our laboratory on L-Alanine (ALA), a glycine-site partial agonist and pregnanolone sulfate (PAS), an allosteric inhibitor with unknown site of action. For these two modulators at concentrations where either drug produces ~50% inhibition, the effect on NMDA receptor responses to physiologic and pathologic patterns of stimulation is modulator-specific and likely to have distinct therapeutic utilities [77,79,80]. 

## 5. Modulators with Stimulus-Specific Effects

By definition, allosteric modulators change the receptor open probability and not the unitary channel conductance. This phenomenon can be definitively demonstrated by recording currents through individual receptors in the presence of the physiological agonists glutamate and glycine (control) and comparing these to activity recorded when the modulator under investigation is also present. Further, these studies can reveal important mechanistic differences between allosteric modulators, differences that may be exploited for therapeutic gain. Here we focus on two allosteric modulators examined under identical conditions at concentrations where each produced ~50% reduction in channel open probability: ALA (1 mM) and PAS (0.1 mM). 

Excitatory effects of alanine on mammalian central neurons were observed very early, during the initial efforts to separate pharmacologically receptor types responsible for excitatory synaptic transmission [85]. A decade later, after learning that glycine is required to observe NMDA-elicited responses from brain neurons, two related aminoacids, alanine and serine were found to substitute effectively for glycine [86]. Although the glycine binding-site on the GluN1extracellular domain represents a quasi-independent unit, the receptor’s affinity for glycine and the potency with which ligands at this site can substitute for glycine depends on the identity of the GluN2 subunit [87]. Thus D-alanine can substitute for glycine with 96% efficacy at receptors containing GluN2A, B or D subunits, but has only 86% relative efficacy at GluN2C-containing receptors. Similarly, l-alanine has relative efficacy that is isoform dependent: it has partial efficacy at receptors containing the two most widely expressed GluN2 isoforms, GluN2A and GluN2B, and full efficacy at the isoforms with more restricted expression, GluN2C and GluN2D [87].

The GluN1/GluN2A (1/2A) isoform is the most prevalent type of NMDA receptor in adult brain. At this receptor isoform, d-alanine is a full agonist at the GluN1 site, having high potency (EC50 = 3 μM) and similar efficacy relative to glycine (98%). In contrast, ALA elicits sub-maximal responses even at saturating concentrations (1 mM, EC50 = 96 μM) and has been categorized as a glycine-site partial agonist [77,87]. Thus for situations where the glycine-site is saturated *in vivo*, ALA, which is competitive with glycine but not with the neurotransmitter glutamate, may represent a therapeutically valuable inhibitor of NMDA receptor mediated responses. 

PAS, a sulfated neurosteroid, directly inhibits NMDA receptors and is noncompetitive with either glutamate or glycine [88,89]. As for ALA, the inhibitory effect is isoform specific and the binding site, although unknown, is most likely located extracellularly [61,90,91]. Importantly, although noncompetitive with either agonist, PAS is use-dependent, being effective only when applied after or simultaneously with the agonists [79,92]. Although both ALA and PAS are allosteric modulators of NMDA receptor-mediated currents their mechanisms are distinct.

**Figure 2:**
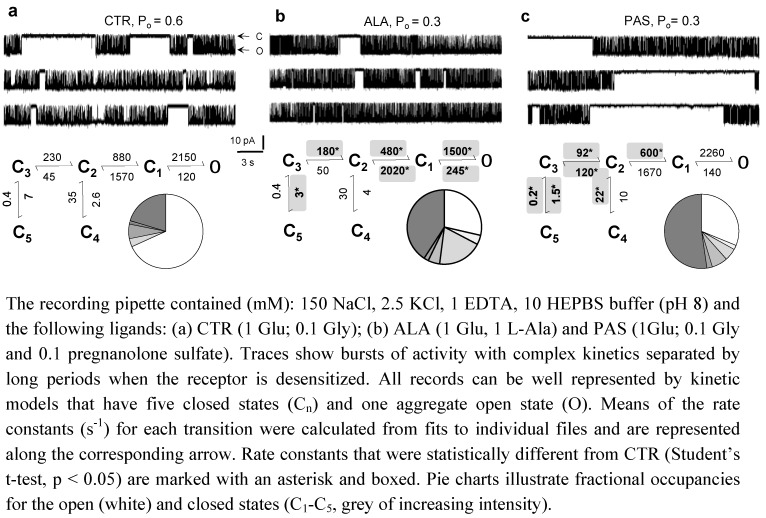
Allosteric modulation of single NMDA receptors. Inward sodium currents were recorded from cell-attached membrane patches of HEK 293 cells having one active 1/2A receptor.

Single-channel studies carried in our lab confirmed previous findings that both ALA and PAS are NMDA receptor modulators with a purely allosteric mechanism [86,92]. Currents were recorded from on-cell membrane patches containing one active 1/2A receptor for tens of minutes with: 1) saturating concentrations of glutamate (1 mM) and glycine (0.1 mM) (control); 2) saturating glutamate and 1 mM ALA; and 3) saturating glutamate and glycine with 0.1 mM PAS (EC_50_ = 63 μM) [79,88,90]. Consistent with an allosteric mechanism, the measured current amplitudes for control (n = 7, 2.7 × 10^7^ events), PAS (n = 7, 8.5 × 10^6^ events) and ALA (n = 6, 9 × 10^6^ events) were not statistical different (p < 0.01) (Figure 2). In contrast and as expected, channel open probabilities were reduced by ~50% relative to control (P_o_, 0.66 ± 0.09) with either ALA (0.27 ± 0.04) or PAS (0.32 ± 0.06) [77,79]. These data fully support earlier indications that ALA and PAS are allosteric inhibitors, and represent definitive evidence for a purely allosteric mechanism. 

Importantly however, ALA and PAS reduced receptor activity with distinct kinetic mechanisms. ALA decreased channel P_o_ by increasing mean closed times and decreasing mean open times: MCT, 14 ± 2 ms *versus* 5.9 ± 1.1 ms, and MOT, 5.3 ± 0.6 ms *versus* 12 ± 0.7 ms, for ALA and CTR, respectively. In contrast, PAS reduced P_o_ exclusively by prolonging closures (32 ± 11 ms) since openings were not statistically different from conrol (10.4 ± 1.1 ms, p < 0.2). All the single-channel files examined originated from one-channel attached patches, and thus we were able to estimate the average frequency with which receptors transitioned from one state to another by fitting kinetic models to the entire sequence of open and closed intervals within each file. This analysis was done with models that represent the activation pathway as a linear sequence initiated by glutamate binding to resting receptors (not shown), which results in the first fully liganded state C_3_, followed by three gating steps, C_3_-C_2_-C_1_-O. The two desensitization reactions observed are represented as separate steps branching off the activation pathway from C_3_ and C_2_. This arrangement allows access into the desensitized states C_5_ or C_4_ only after agonists are bound, and links the receptor’s bursting frequency with its recovery from desensitized states (Figure 2).

This analysis allowed us to further dissect the differences in mechanism initially inferred from dwell time distributions. Results showed changes that were allocated broadly across several rate constants for ALA but were more localized for PAS (Figure 2). ALA behaved similarly to other glycine- and glutamate-site partial agonists, a strong indication that structural changes at agonist binding-sites reverberate kinetically across multiple sequential transitions [77,80]. However, these changes were principally observed at kinetic steps occurring along the activation pathway, and not for transitions into desensitized states. In contrast, the changes produced by PAS were restricted to the initial portion of the gating sequence (C_3_-C_2_) and were pronounced for the C_3_-C_5_ and C_2_-C_4_ steps associated with entry into and recovery from desensitized states. Thus with kinetic analyses of single-channel traces we were able to identify the kinetic steps most sensitive to each modulator and observed that the pattern of change was modulator-specific.

**Figure 3:**
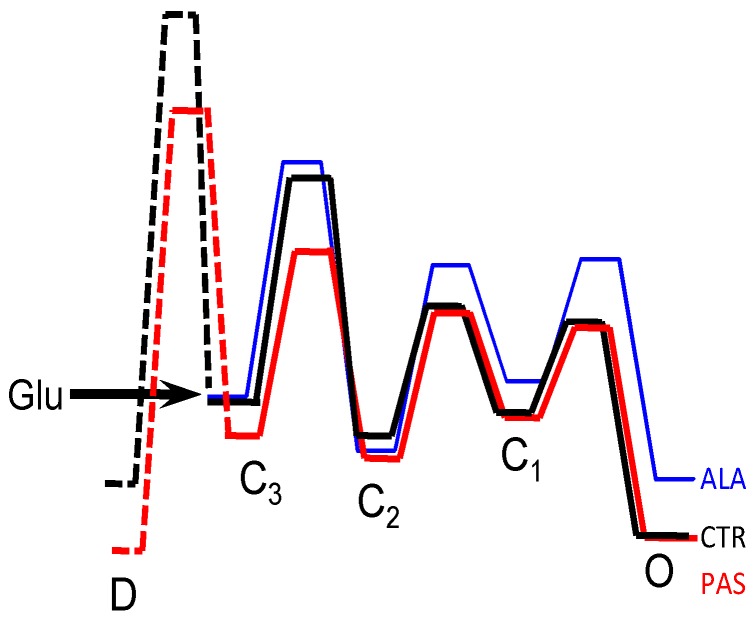
Free energy profiles of NMDA receptors during stationary gating. Energy landscapes were constructed as described in the Experimental Section, using the rate constants illustrated in Figure 2. Energy fluctuations were calculated relative to the first glutamate-bound state C_3_ (arrow) for all models; PAS-bound diagram was aligned with CTR diagram at the open states, based on experimentally determined equal open durations in these conditions.

Clearly, these results would be more informative if the structural correlates of the C and O states were known. However, even in the absence of this knowledge, important inferences can be made by considering the functional properties of the kinetic states postulated by the model. For example, using the rates estimated by fits to single-channel records, the model predicts that at equilibrium PAS-bound receptors will reside in desensitized states with higher probabilities as compared to ALA-bound receptors, even though their open state occupancies are similar (Figure 2, pie chart). This prediction can be tested and may have valuable consequences for drug design (see below). In addition, the model can be used to calculate free energy profiles and construct visual representations of the different energetic differences elicited by ALA and PAS binding (Figure 3).

As a first-pass test for these predictions we examined whether the model, which was deduced from equilibrium single-channel measurements, can reproduce correctly non-stationary ensemble responses. First, we used the models in Figure 2 to calculate the time-dependent accumulation of receptors in open states following prolonged (5 s) exposure to saturating concentrations of glutamate (1 mM).

**Figure 4:**
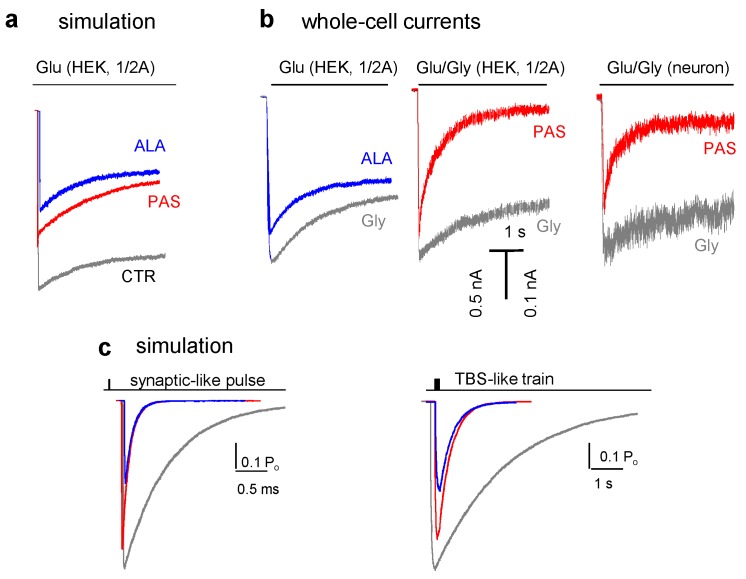
Effect of allosteric modulators on ensemble NMDA receptor responses to physiologic and pathologic patterns of glutamate exposure. (a), (c) Macroscopic current traces were simulated using the models illustrated in Figure 2 and a similar number of receptors (100 receptors) after appending two sequential glutamate-binding steps to the first liganded state C3 (see Experimental section). PAS binding was modeled from O state of glutamate bound receptors [79]. Exposure to 1 mM glutamate mimicked: a synaptic pulse (1 ms), a theta-burst stimulus (TBS, five 1-ms pulses delivered at 100 Hz), and pathologic exposure (continuous). (b) Whole-cell current traces were recorded in the presence of 0.1 mM Gly following 5-s pulses of 1 mM Glu from 1/2A receptors expressed in HEK 293 cells and from cultured cortical neurons (with CNQX in the recording pipette to inhibit AMPA receptor responses).

This protocol most sensitively detects changes in receptor desensitization and can inform about modulator effects on NMDA receptor currents during prolonged exposure to glutamate, situation that most likely occurs during excitotoxic episodes. Results predict that both ALA and PAS will be equally effective in reducing NMDA receptor responses to prolonged glutamate exposure (Figure 4a). This prediction was validated by whole-cell NMDA receptor responses measured form recombinant 1/2A receptors expressed in HEK 293 cells and from receptors native to cultured rat cortical neurons (Figure 4b) [77,79]. 

Because both modulators reduce NMDA receptor elicited currents by continued exposure to glutamate, these results suggested that both ALA and PAS would limit NMDA receptor-mediated calcium entry, and perhaps may limit the damaging effects of uncontrolled extracellular glutamate concentrations that occur for example following hypoxic ischemia, traumatic brain injury or in chronic neurodegenerative disorders. Consistent with these assumptions, pregnanolone hemisuccinate, a pregnanolone derivative which has higher water solubility than PAS, reduced cortical and sub-cortical infarct size in a mouse model of focal ischemia [93]. However, our results suggest that ALA is unlikely to have similar effects. Although relative to glycine-elicited currents, ALA inhibits substantially NMDA receptor currents, it will potentiate currents from receptor populations whose glycine-binding sites are not saturated. This appears to be the case in several brain regions [42]. 

The kinetics of NMDA receptor-mediated currents changes with time after whole-cell access and after patch excision, indicative of alterations in receptor gating due to the associated disruption in physiological intracellular milieu [36,94,95,96]. Still, even if exact matches between cell-attached model predictions and currents recorded from whole-cell or excised patch preparations cannot be expected, the model should predict correctly the pattern of modulator-dependent kinetic change, regardless of preparation. Thus the result that both ALA and PAS increased desensitization of measured whole-cell currents as predicted by the models derived from single-channel analyses gives further credibility to these models and encourages their use as a first-pass *in silico* assessment of modulator safety and tolerability. For example, modulator effects on normal synaptic transmission can be examined *a priori* by examining modulator-dependent changes on responses to physiological patterns of stimulation.

When applied to receptors experiencing brief synaptic-like stimulation, our models predict that ALA and PAS will have very different effects on receptor responses. Figure 4c illustrates model predictions for situations where the receptors are exposed to a single 1-ms pulse of 1-mM glutamate or to a burst of five such pulses at 100 Hz frequency. These patterns of glutamate release occur physiologically during low- or high-frequency stimulation, and may offer clues about how modulators affect normal synaptic transmission. The models predict that PAS will have negligible effects on the NMDA receptor response amplitude to low frequency stimulation and minimal effect on the theta-burst elicited response, whereas ALA reduced responses by ~50% [77]. These results suggest that normal synaptic transmission will proceed largely unchanged when PAS is present at concentrations that cut in half responses from receptors continually exposed to high concentrations of glutamate, such as may be the case with extrasynaptic receptors in pathologic situations. The ability to allow normal transmission to proceed while inhibiting pathologic activations is a very desirable feature for a neuroprotective agent and thus, PAS deserves closer examination as a potential NMDA receptor therapy. In contrast, because ALA is competitive with the physiologic agonists glycine and D-serine, *in vivo* concentrations of glycine will significantly influence the outcome on receptor responses. In this respect, our results highlight the need to more clearly delineate physiologic levels of glycine and D-serine and their regulation in health and disease.

Clearly, *in silico* predictions can only serve as starting points in mechanistic investigations of allosteric modulators with therapeutic potential. Still, the results summarized here argue that even when two allosteric modulators alter equilibrium NMDA receptor currents to the same level, the mechanism employed by each has important consequences on both therapeutic efficacy and tolerability. It is important to keep in mind that most known channel modulators were evaluated by measuring modulator-induced changes in the amplitude of currents recorded from channels expressed in frog oocytes. This approach while convenient, sensitive and relatively high throughput provides little if any information about kinetics and thus mechanism. We hope that the results presented here emphasize the requirement for more detailed mechanistic investigation of even the known modulators, aside from the uncontested need to search for new compounds that act at known sites, and of altogether novel sites. 

## 6. Conclusions

NMDA receptors are critical to myriad brain functions and also culprits in a number of severe neuropathologies. Despite the so far minimal success of NMDA receptor targeted therapies, allosteric NMDA receptor modulators remain attractive candidates in the quest for pharmaceuticals that will alleviate suffering from mental disorders. To identify modulators that are both effective and safe, substantial advances must occur in several areas. First, it will be important to delineate how information encoded in NMDA receptor fluxes contributes to specific brain functions. Second, high-resolution descriptions of functional domains and modulator sites for the principal NMDA receptor isoforms remains a high priority. Finally, detailed kinetic mechanisms for each modulator/receptor isoform pair will provide the tools necessary to make informed decisions as to which pairs to pursue for each dysfunction. This knowledge will be tremendously powerful in identifying rational approaches to both therapeutic strategy and drug design, and in building an arsenal with specific weapons for each disease, and ideally for every patient. 

## Abbreviations

ACBC: aminocyclobutane-1-carboxylic acid, GluN1 partial agonist;
ACPC: 1-aminocyclopropane-1-carboxylic acid, GluN1 partial agonist;
ALA: l-alanine, GluN1 ligand;
AMPA: alpha-amino-3-hydroxyl-5-methyl-4-isoxazole-propionic acid;
APV: α-amino-5-phosphonopentanoic acid, GluN2 antagonist;
DCKA: 5,7-dichlorokynurenic acid, GluN1 antagonist;
DCS: d-cycloserine, GluN1 ligand;
PAS: pregnanolone sulfate, 3α-hydroxy-5β-pregnan-20-one sulfate
